# Antibiotic Discontinuation at Clinical Resolution Versus Seven Additional Days for Canine Superficial Pyoderma: A Randomized Clinical Study

**DOI:** 10.3390/vetsci13090880

**Published:** 2026-08-28

**Authors:** Jennifer L. Clegg, Clarissa P. Souza, Sheila M. F. Torres

**Affiliations:** 1 Department of Veterinary Clinical Medicine, College of Veterinary Medicine, University of Illinois at Urbana-Champaign, Urbana, IL 61802, USA; jennagvet82@gmail.com; 2 Southwest Veterinary Dermatology, Houston, TX 77070, USA; 3 Department of Veterinary Clinical Sciences, College of Veterinary Medicine, University of Minnesota, Saint Paul, MN 55108, USA

**Keywords:** antibiotic resistance, antibiotics, dogs, stewardship, superficial pyoderma

## Abstract

The primary objective of this study was to determine if superficial pyoderma (SP) recurs sooner and at a higher rate in dogs not treated beyond clinical resolution. Sixty-seven client-owned dogs were randomly assigned to either group A (*n* = 37), treated with a culture-based oral antimicrobial for seven days beyond resolution, or to group B (*n* = 30), treated until resolution. After withdrawals, 60 dogs were analyzed for SP resolution. Dogs were examined every 14 days until D98, unless excluded due to unresolved SP by D42 or if SP recurred before D98. At each visit, a dermatologic exam and pyoderma severity score determination (0–21) were performed. Clinical resolution of SP was observed in 28 days (median) in 20/33 dogs in group A and in 18/27 in group B and not observed by D42 in 13/33 dogs in group A and 9/27 in group B. These dogs were considered treatment failures. SP recurred in 13/20 dogs in group A and 10/18 in group B after 67.5 days and 69 days (median) of follow-up time in group A and B, respectively. The median follow-up time to SP recurrence was 67.5 days and 69 days in group A and B, respectively. The hazard ratio for the risk of SP recurrence in group A compared to group B was 1.04 (95% CI 0.42–2.66; *p* = 0.9319). In this sample, there was no statistically significant difference between the groups for pyoderma score, resistance patterns, time to SP resolution, time to recurrence, recurrence rate, or probability of recurrence. Extending antibiotics seven days beyond clinical resolution is not necessary for canine SP treatment.

## Simple Summary

The primary objective of this study was to determine if superficial pyoderma (SP) recurs sooner and at a higher rate in dogs not treated beyond clinical resolution. Sixty-seven client-owned dogs were randomly assigned to either group A (37), treated with a culture based oral antimicrobial for seven days beyond resolution, or to group B (30), treated until resolution. After withdrawals, 60 dogs were analyzed for SP resolution. There was no statistically significant difference between the groups for pyoderma score, resistance patterns, time to SP resolution, time to recurrence, recurrence rate, or probability of recurrence. Extending antibiotics seven days beyond clinical resolution is not necessary for canine SP treatment.

## 1. Introduction

Superficial pyoderma (SP) is a bacterial infection involving the epidermis and follicular epithelium. It is a commonly diagnosed dermatologic condition in dogs and one of the main clinical presentations leading to systemic antibiotic use in small-animal practice [1,2,3]. Different bacteria can be commensals or opportunistic pathogens and can cause SP in dogs. *Staphylococcus pseudintermedius* is a coagulase-positive bacterium primarily associated with SP in dogs. However, less commonly *S. coagulans* (formerly *S. schleiferi* subsp. *coagulans*) [4] and other bacteria are also isolated from SP cases [3,5,6,7].

Most cases of SP in dogs are secondary to an underlying disease that predisposes the development of skin infections. The primary disease triggering SP must be identified and managed to avoid SP recurrence and repeated antimicrobial treatment. Recently, concern over antimicrobial resistance has been increasing within the veterinary medical community. Superficial pyoderma treatment has become challenging due to the evolving recognition of methicillin-resistant *S. pseudintermedius* and multidrug-resistant (MDR) *Staphylococcus* amongst canine clinical isolates [6,7,8]. Frequent and long-term antibiotic use can increase selection pressure for resistance among commensal bacteria and have significantly contributed to the selection and transmission of resistant bacteria [9,10,11].

Historically in human medicine, antibiotic courses were set by fear of under-treatment, with less concern about overuse. However, growing evidence is showing that antibiotic courses shorter than conventionally recommended are often appropriate [12,13,14]. Therefore, the advice to patients to complete a course of treatment after clinical signs have been resolved has been questioned [12,15,16,17]. Different studies indicate that short-term strategies have equivalent clinical outcomes to longer courses and are associated with lower rates of infection recurrence and antibiotic resistance [17,18]. Shorter courses of antibiotics can also avoid collateral selection, which happens when a patient is being treated for any reason and the commensal flora of antibiotic sensitive bacterial strains on their skin or gut are replaced by resistant ones, distant from the site of the initial infection [15,17,19].

Veterinary practice guidelines of prudent antibiotic use are negatively affected by numerous knowledge gaps, including optimal duration of treatment [20,21]. Commonly prescribed durations of antibiotic therapy for bacterial skin infections are not evidence-based, and recommendations for SP remain controversial. Previous recommendation by the International Society for Companion Animal Infectious Diseases (ISCAID), based only on clinical experience, was to extend systemic antibiotic therapy by one week beyond clinical resolution [2,21,22]. The current ISCAID guidelines revise outdated recommendations and propose shorter antimicrobial courses based on drug approval trial data from U.S. Food and Drug Administration Freedom of Information and human medicine evidence [3]. Therefore, research is needed to determine if canine SP can be treated with shorter antibiotic courses.

The primary objective of this study was to determine the probability of SP recurrence and if SP recurs sooner and at a higher rate in dogs not treated beyond the clinical resolution of infection. This information can contribute evidence-based data to evaluate whether extending systemic antibiotic therapy for SP is a justified recommendation. The secondary study objectives included the following: (i) to determine the average time for SP to resolve in dogs; (ii) to investigate if the clinical severity of infection correlates with the bacteria resistance pattern at enrollment; (iii) to compare the susceptibility pattern of the bacterium isolated at the time of study enrollment and infection recurrence; and (iv) to investigate if the bacterium isolated at enrollment, the systemic antibiotic prescribed, and the cAD treatment correlate with the time for SP resolution and SP recurrence.

## 2. Materials and Methods

### 2.1. Ethics

The study was approved by the Institutional Animal Care and Use Committee (IACUC #20070 and #23077), and owner consent forms were signed upon enrollment.

### 2.2. Inclusion Criteria

Dogs of any age, sex, or breed diagnosed with SP based on clinical signs and the presence of coccoid bacteria and degenerate neutrophils on cytology were enrolled. A complete medical history of each patient was obtained at enrollment.

### 2.3. Exclusion Criteria

Dogs were not enrolled if one of the following were present: (i) clinical lesions and cytology results compatible with deep pyoderma; (ii) *Malassezia* dermatitis confirmed by cytology; or (iii) inability to receive systemic antibiotic therapy for any reason.

### 2.4. Withdrawal Criteria

Dogs were withdrawn if any of the following occurred: (i) lack of owner compliance with study protocol; (ii) adverse events or medication intolerance; and (iii) development of *Malassezia* dermatitis while the SP was cytologically resolved, preventing clinical resolution of lesions during the study period.

### 2.5. Concurrent Treatments

All enrolled dogs had a previous diagnosis of canine atopic dermatitis (cAD; food and/or environmentally induced). Systemic medications to control cAD clinical signs that started at least two weeks prior to enrollment were allowed to continue if unchanged during the study period. All dogs were maintained on appropriate heartworm, flea, and tick prophylaxis throughout the study. No diet changes were instituted, and topical antimicrobial therapy was not allowed during the study period.

### 2.6. Bacterial Aerobic Culture and Susceptibility Testing

At the time of enrollment, Day (D) 0, each dog had its SP lesions sampled for aerobic bacterial culture and susceptibility testing. Samples were collected with a sterile swab from representative SP lesions (intact pustules if present, epidermal collarettes, and/or papules) and submitted to an accredited veterinary microbiology laboratory. Based on the susceptibility results and clinician discretion, dogs were prescribed a systemic antibiotic at recommended dosages for SP. Moreover, if the SP recurred during the study period, the aerobic bacterial culture and susceptibility test were repeated. Based on the susceptibility results and clinician discretion, dogs were prescribed a systemic antibiotic at recommended dosages for SP.

### 2.7. Treatment Groups

Dogs were randomly assigned to two groups using a computer-generated list (www.graphpad.com). Group A received a systemic antibiotic for seven days beyond the complete resolution of SP lesions, and group B received a systemic antibiotic until complete resolution of SP lesions.

### 2.8. Clinical and Cytological Evaluations of Superficial Pyoderma

A pyoderma severity score was performed to evaluate SP lesion extent. The body was divided into seven areas, including dorsal trunk, lateral trunk, axillae, ventral thorax, ventral abdomen, inguinal area, and inner thighs. Erythematous papules, pustules, crusts, epidermal collarettes, and moth-eaten alopecia were considered characteristic SP lesions. Lesion severity at each affected area was determined by using the following scoring system: 0 (no lesion), 1 (mild—less than 25% of the affected area), 2 (moderate—25–50% of the affected area), and 3 (severe—>50% of the affected area). The maximum achievable lesion severity score was 21 (i.e., 7 × 3). Pododermatitis in dogs with cAD is commonly caused by deep pyoderma and/or *Malassezia* infection [23]; therefore, we opted to exclude this from clinical evaluation. Cytology of lesions was performed at the time of enrollment (D0) and at each recheck visit if SP lesions were still observed.

### 2.9. Assessment of Superficial Pyoderma Resolution and Recurrence

Dogs were consistently evaluated by the same investigator, a veterinary dermatology resident, supervised by a board-certified veterinary dermatologist at 14-day intervals until D98 (end of the study) or until SP recurrence, whichever occurred first.

**Figure 1:**
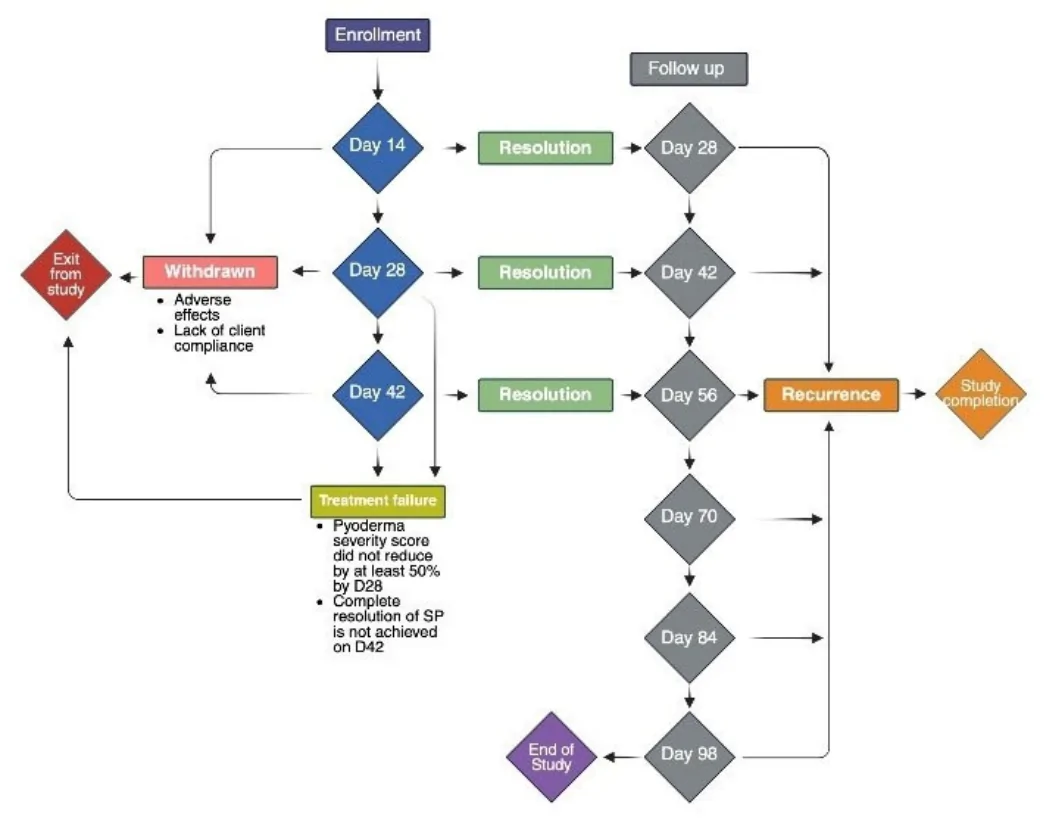
This flowchart illustrates the progression of the study, with diamonds representing key visits and decision points. The blue diamonds (Days 14, 28, and 42) mark examination time points whilst receiving systemic antibiotic therapy. The grey diamonds (Days 28, 42, 56, 70, 84, and 98) indicate follow-up examinations after superficial pyoderma resolution. The red diamond represents a withdrawal from the study due to a lack of client compliance or the development of adverse events. Treatment failure was determined on Day 28 or Day 42 according to study criteria. The orange diamond marks the completion of the study, with a recurrence of superficial pyoderma occurring before the study’s conclusion on Day 98. The purple triangle represents the end of the study period. Created in BioRender. https://BioRender.com/e58h652, accessed on 4 January 2025.

Resolution of clinical signs was defined as the complete absence of SP lesions. If less than a 50% reduction in total pyoderma score was observed by D28, treatment failure was considered, and lesions were sampled for another aerobic bacterial culture and susceptibility test. Topical treatment was prescribed for the remaining SP. If a greater than 50% reduction in total pyoderma score was observed by D28 without complete resolution, the dog continued in the study and was given antibiotics until D42. Treatment failure was determined at D42 if SP was not completely resolved, prompting the same measures above. Dogs with SP resolution by D42 were monitored for recurrence until D98. Superficial pyoderma recurrence was defined by the return of characteristic SP lesions and by supporting cytological findings. Figure 1 illustrates the study flowchart.

### 2.10. Statistical Methods

The sample size was calculated using R (R Core Team 2018) based on a significance level of 0.05, a power of 0.8, and a standard deviation of 10. To detect differences in recurrence times between the study groups of one week or more, 32 patients need to be enrolled for each group (total = 64 dogs). Survival analysis was conducted to determine whether SP recurs sooner in dogs not treated beyond clinical resolution. Kaplan–Meier curves evaluated the median follow-up time to SP resolution in 60 dogs and SP recurrence in 38 dogs and were generated to determine any difference in time to recurrence between the groups. Log-rank tests statistically compared the survival curves between the groups including 38 dogs. The median follow-up time, defined as the number of days from the resolution of SP to recurrence, with dogs not experiencing a recurrence during the study period considered censored, was calculated for 38 dogs. Cox proportional hazards analyzed the risk of recurrence between groups including 38 dogs. A Fisher’s exact test evaluated the frequency of SP resolution in 60 dogs and recurrence of 38 dogs between the groups. A *t*-test evaluated the mean of pyoderma severity scores of 60 dogs along with time to resolution of 38 dogs and recurrence between the groups including 38 dogs. Cox proportional hazards modeling analyzed factors influencing the follow-up time and recurrence rate of 38 dogs, with likelihood ratio tests assessing covariate significance with 95% confidence intervals for the hazard ratios. Pearson correlation assessed the correlation between the pyoderma severity score and time to resolution in 38 dogs, lack of resolution in 22 dogs, and recurrence rate in 38 dogs. The Kruskal–Wallis test explored the relationship between the pyoderma severity score and bacterial resistance pattern, as well as the association between the bacterial isolate group at enrollment, bacterial resistance patterns, cAD treatment, and antibiotic therapy with the time to SP resolution in 38 dogs, recurrence in 38 dogs, or treatment failure in 22 dogs.

For the statistical analyses, isolates from aerobic bacterial culture were classified as follows: (i) *Staphylococcus* sp.; (ii) *Staphylococcus* sp. + one or more coccus bacteria; (iii) *Staphylococcus* sp. + rod bacteria; or (iv) *Staphylococcus* sp. + another coccus bacteria + rod bacteria (Table S1). The resistance patterns were determined based on the *Staphylococcus* isolates. These patterns were reported as methicillin susceptible (MS), methicillin resistant (MR), MDR, or extensively drug resistant (XDR). A *Staphylococcus* isolate was classified as MDR if resistant to three or more classes of antibiotics and at least one antibiotic in each class was reported intermediate or resistant on susceptibility testing; it was classified as XDR if susceptible to only one or two classes of antibiotics. Dogs withdrawn from the study were not included in the statistical analysis. All statistical analyses were conducted using SAS software 9.4v (SAS Institute Inc., Cary, NC, USA), with a significance level of *p* < 0.05.

## 3. Results

### 3.1. Study Population

From October 2022 to September 2024, a total of 67 client-owned dogs were enrolled, with 37 in group A and 30 in group B.

Group A included 24 male and 13 female dogs at the ages of 0.75 to 14 years (mean 5.65 ± 3.49). Breeds included mixed breed (16), American pit bull terrier (3), English springer spaniel (2), Great Dane (2), Labrador retriever (2), Yorkshire terrier (2), American Staffordshire terrier (1), Boston terrier (1), boxer (1), cocker spaniel (1), English bulldog (1), golden retriever (1), German Shepherd (1), Olde English bulldogge (1), Shetland sheepdog (1), and shih tzu (1).

Group B included 21 male and 9 female dogs at the ages of 1 to 15 years (mean 5.25 ± 3.5 years). Breeds included mixed breed (10), cocker spaniel (3), Labrador retriever (3), English bulldog (2), English foxhound (2), German Shepherd (2), American bulldog (1), American pit bull terrier (1), Australian shepherd (1), boxer (1), Great Dane (1), miniature schnauzer (1), Pomeranian (1), and Shetland sheepdog (1).

**Figure 2:**
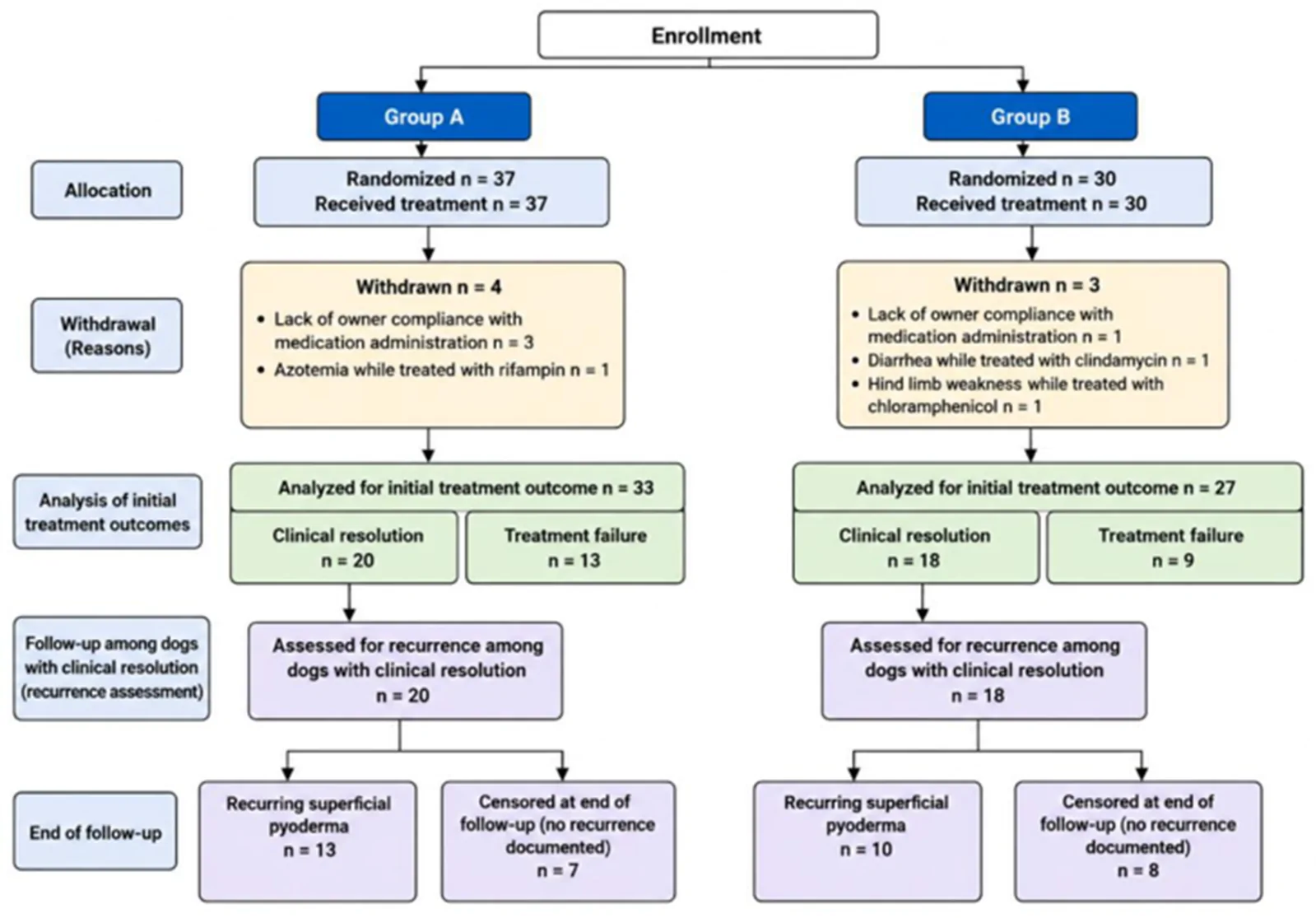
Consort flow diagram. CONSORT (Consolidated Standards of Reporting Trials).

Figure 2 shows a flowchart of enrolled dogs through different stages of the study.

Table 1 includes the frequency of the prescribed antibiotic and treatment for cAD by group and bacterial isolate at enrollment.

Seven dogs (four in group A and three in group B) were withdrawn from the study for lack of owner compliance with antibiotic instructions or development of adverse events associated with antibiotic administration. Four other dogs were diagnosed with *Malassezia* dermatitis and a non-resolved SP on D42. These four dogs were not considered withdrawn cases and were analyzed as SP treatment failures.

### 3.2. Superficial Pyoderma Severity Scores at Enrollment

The mean pyoderma severity scores at enrollment did not differ between the groups. Dogs in group A had a mean pyoderma score of 7.92 ± 3.73, and group B scored 7.77 ± 3.87 (95% CI −1.75–2.05; *p* = 0.8727). The minimum and maximum scores for each group ranged from 1 to 16.

There was no statistically significant difference in the pyoderma severity scores across the different resistance patterns of the *Staphylococcus* isolates at enrollment (*p* = 0.7).

### 3.3. Resistance at Enrollment

Resistant Staphylococcus isolates were identified in 22 dogs at enrollment. Sixteen of twenty-two (72.8%) dogs, ten in group A and six in group B, received at least one course of systemic antibiotic in the year prior to enrollment. Six out of the sixteen (37.5%) dogs, all in group A, received systemic antibiotics in the month prior to enrollment. Susceptible Staphylococcus isolates were identified in 45 of 67 dogs at enrollment; 26 of 45 (57.8%) dogs, 14 in group A and 12 in group B, had at least one course of systemic antibiotic within a year prior to enrollment. Three of the twenty-six (11.5%) dogs from group A received a systemic antibiotic in the month prior to enrollment.

### 3.4. Superficial Pyoderma Resolution

**Figure 3:**
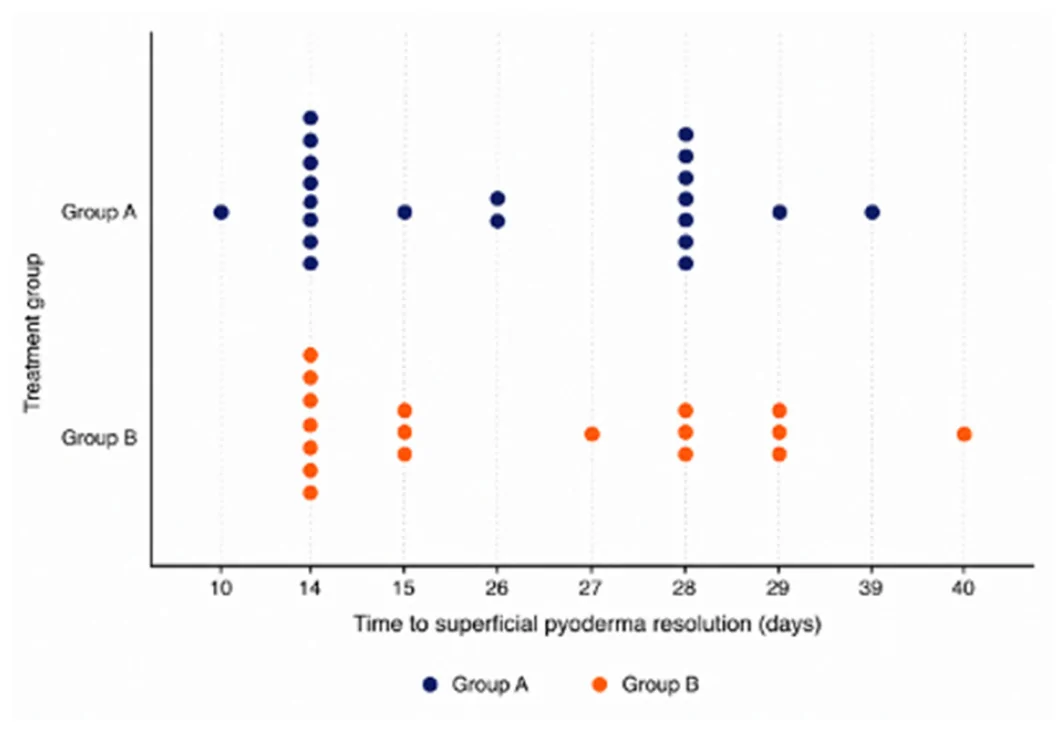
Dot plot depicting the number of dogs achieving superficial pyoderma resolution on each study day for group A and group B. Each dot represents an individual dog. Group A dogs were treated with systemic antibiotics for seven days beyond clinical resolution, while group B dogs were treated until clinical resolution.

Superficial pyoderma resolution was observed in 20 of 33 (60.6%) dogs in group A and 18 of 27 (66.7%) dogs in group B. There was no statistically significant difference in the frequency of SP resolution between the groups (95% CI 0.59–1.37; *p* = 0.8). Based on Kaplan–Meier survival estimates, the follow-up time to resolution was 28 days (median) in groups A (95% CI 28 days to not estimable) and B (95% CI 15–29 days). The time to resolution ranged from 10 to 39 days in group A and from 14 to 40 days in group B. A one-to-four-day time period before or after the fourteen-day recheck interval was accepted to accommodate owners’ schedules and holidays. There was no statistically significant difference between groups for time to SP resolution (*p* = 0.7834). Figure 3 illustrates the frequency of dogs by group at various SP resolution time points.

The cAD treatment (*p* = 0.3574), the bacterium isolated at enrollment (*p* = 0.1773), and the antibiotic prescribed (*p* = 0.3802) were not significantly associated with the time for SP resolution. Moreover, among the dogs with SP resolution, there was no statistically significant difference in the time to resolution across the different resistance patterns at enrollment (*p* = 0.853).

Although not reaching statistical significance (95% CI 1.021–15.676; *p* = 0.0654), MDR status was associated with a 3.449 hazard ratio for faster SP resolution compared to XDR cases. Due to the low number of cases in the *Staphylococcus* + cocci group (one dog), it was not possible to analyze the association between bacterial groups at enrollment and the SP resolution.

### 3.5. Superficial Pyoderma Recurrence

After SP resolution, 7 out of 20 (35.0%) dogs in group A and 8 out of 18 (44.4%) dogs in group B did not experience SP recurrence by D98. The SP in these dogs remained controlled for 81.29 ± 5.31 and 76.63 ± 10.0 days in groups A and B, respectively.

Superficial pyoderma recurrence was observed prior to D98 in 13 out of 20 (65.0%) dogs in group A and 10 out of 18 (55.6%) in group B. The SP recurrence rate between groups was not significantly different (95% CI 0.70–1.97; *p* = 0.74). Including only dogs that experienced SP recurrence, the mean time was 45.31 days in group A and 29.3 days in group B. There was no statistically significant difference between groups for the time of SP recurrence (95% CI −4.63–36.65; *p* = 0.12).

The cAD treatment (*p* = 0.7974), the bacterium isolated at enrollment (*p* = 0.5659), and the antibiotic prescribed (*p* = 0.2738) were not significantly associated with time to SP recurrence.

At recurrence, the bacterial isolates and *Staphylococcus* resistance patterns by group are included in Table 2. Of dogs with SP recurrence, bacteria and resistance patterns at enrollment and SP recurrence are included in Supplementary Tables S2 and S3.

**Figure 4:**
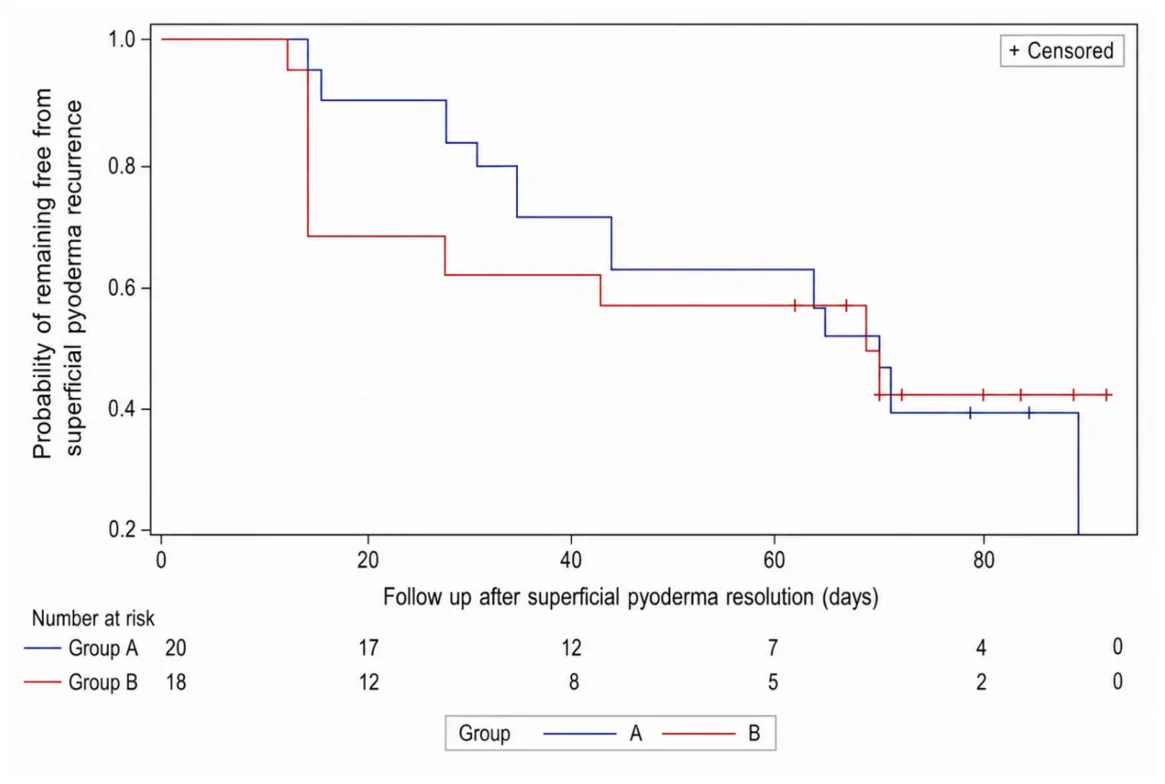
A Kaplan–Meier curve was evaluated to determine the time to SP recurrence and was generated to determine any difference in time to recurrence between group A (treated seven days beyond clinical resolution) and group B (treated until clinical resolution). Day 0 indicates the day of superficial pyoderma resolution and the start of post-resolution follow-up. Censored data included dogs without superficial pyoderma recurrence by the end of the study period (Day 98), which are indicated by + signs on the curve.

The median follow-up time from SP resolution until the end of the study, which accounted for dogs that experienced SP recurrence and censored dogs, was 67.5 in group A, ranging from 14 to 88 days, and 69 days in groups B, ranging from 12 to 70 days. A Kaplan–Meier survival curve was performed to evaluate the probability of SP recurrence after initial resolution in each group (Figure 4). Each vertical drop in the line occurs each time a dog in the corresponding group experiences a recurrence of SP. The magnitude of the drop corresponds to the proportion of dogs affected at that time point. A log-rank test showed no significant difference between groups (*p* = 0.9439). The hazard ratio for the risk of SP recurrence in group A compared to group B was 1.04 (95% CI 0.42–2.66; *p* = 0.9319).

Although not statistically significant (95% CI 0.26–10.11; *p* = 0.444), the hazard ratio for SP recurrence was 1.982 when comparing mixed infections (*Staphylococcus* + cocci + rod isolates) to *Staphylococcus* sp. alone. There was no significant difference in the time to recurrence across the different resistance patterns at enrollment (*p* = 0.523).

### 3.6. Superficial Pyoderma Treatment Failure

SP resolution was not achieved by D42 in 13 out of 33 (39.4%) dogs in group A and 9 out of 27 (33.3%) dogs in group B. Moreover, 8 out of 13 (61.5%) dogs in group A and 5 out of 9 (55.5%) in group B that failed to respond to antibiotic treatment by D28 exited the study and had a repeated culture. The bacterial isolates from group A dogs were five (62.5%) *Staphylococcus* sp. and three (37.5%) *Staphylococcus* + rod, and their resistance patterns were reported as two (25.0%) MDR, three (37.5%) XDR, and three (37.5%) susceptible to all antibiotics tested. The bacterial isolates from group B dogs were three (60.0%) *Staphylococcus* sp. and two (40.0%) *Staphylococcus* + rod, and their resistance patterns were reported as three (60.0%) susceptible to all antibiotics tested, one (20.0%) MRS, and one (20.0%) XDR.

Of these dogs, bacteria and resistance patterns at enrollment by dog and group are included in Table S4. Possible reasons attributed to the SP treatment failure were as follows: (i) a lack of systemic antibiotic response in 12 out of 13 (92.3%) dogs in group A and 6 out of 9 (66.7%) dogs in group B and (ii) a lack of systemic antibiotic response in combination with the development of yeast dermatitis in 1 out of 13 (7.7%) dog in group A and 3 out of 9 (33.3%) dogs in group B.

The type of cAD treatment (*p* = 0.5323), the type of antibiotic prescribed (*p* = 0.6368), and the bacterial resistance pattern at enrollment (*p* = 0.539) were not significantly associated with the SP treatment failure. Individual dog data, including days to SP resolution, SP recurrence, recurrence-free interval, prescribed antibiotics, and concurrent medications with dosages and administration frequencies, are provided in Supplementary Tables S5–S8.

## 4. Discussion

Our study evaluated if the continuation of systemic antibiotic therapy for seven days beyond SP clinical resolution is a valid rule of thumb for dogs. Conventionally, it has been recommended to treat dogs for an additional week after SP clinical resolution, although the evidence for this was largely anecdotal [22]. The most recent antimicrobial use guidelines for canine pyoderma recommend the discontinuation of systemic antibiotic upon resolution of clinical signs of SP. The authors consider that the historical suggested treatment duration is no longer justified [3], although specific supportive data are not available. In our study, when comparing dogs treated with antibiotics for one week beyond SP resolution to those treated only until SP resolution, there was no statistically significant difference in the probability of SP recurrence or recurrence rate, indicating that discontinuing antibiotic therapy at the time the SP resolves does not increase the risk for recurrence. The current knowledge shows that it is essential to identify the optimal length of systemic antibiotic therapy in cases of canine SP because shorter antibiotics courses reduce selection pressure for bacterial resistance, may improve client compliance, cost less, and decrease adverse medication events [8,22].

The median follow-up time to SP resolution among studied dogs in both groups was 28 days, with 10 dogs in group A and 11 dogs in group B showing SP resolution within 14 days of treatment. Our findings corroborate with previous references of treatment duration for SP resolution varying from 14 to 42 days [2,24,25,26]. Superficial pyoderma resolution was observed on D10 in one studied dog. This dog was rechecked earlier due to the owner’s schedule. It is possible that some other dogs also had SP resolved sooner than their first recheck visit planned for D14. In human medicine, it has been discussed that the concept of an antibiotic course ignores the fact that patients may respond differently to the same antibiotic, depending on diverse patient and disease factors. Patients may be advised to stop treatment when they feel better [17]. This recommendation may not be as feasible with veterinary patients as owners may not be capable of recognizing remaining clinical signs of infection. However, it should encourage veterinarians to have more frequent treatment response assessments by increasing client communication and scheduling earlier rechecks.

Defined outcome measures for SP resolution in dogs are not available in the literature. In our study, we considered treatment failure when the pyoderma severity scoring system did not decrease by at least 50% by day 28 or if the lesions were not completely resolved by D42. A large number of dogs did not respond to the systemic antibiotic course despite the culture-based prescription and were considered treatment failures. Similar outcomes have been previously reported where systemic antibiotic was administered to dogs with SP, although empirically prescribed, and showed only partial improvement or complete lack of SP resolution with worsening lesions [24,26]. Systemic antibiotic efficacy not only depends on bacterial susceptibility, but it is also influenced by the drug administration, appropriate dosing, severity of infection, and management of underlying diseases [7].

Topical antimicrobial therapies have been commonly prescribed for the treatment of canine SP, alone or combined with systemic antibiotics [7,27,28,29]. In our study, topical antimicrobials were prohibited to avoid confounding results, as we specifically aimed to evaluate systemic antibiotic use. It is possible that the time for SP resolution could have been reduced in some dogs if a topical antimicrobial had been added to the protocol, or even dogs that failed to respond to systemic antibiotics could have had a different outcome. We speculate that SP recurrence might have been delayed with concurrent topical therapy. Although lacking supportive literature, topical antimicrobials are also routinely used as maintenance therapy to limit the recurrence of SP in dogs [30].

Cephalexin, amoxicillin + clavulanic acid, and clindamycin are first-choice antibiotics recommended for the empirical treatment of SP in dogs [3]. Although an aerobic culture was performed from SP lesions of enrolled dogs for the purpose of the study, the prescription of these first-choice antibiotics was still preferred, when possible, based on the susceptibility results. Other antibiotics, including chloramphenicol, trimethoprim + sulfa, rifampicin, and marbofloxacin, were only prescribed if a first-choice antibiotic was not an option. Vancomycin and linezolid were not considered options for the treatment of SP in dogs, as these medications are deemed critically important to human health [22].

In our study, the bacterial resistance at enrollment was not associated with longer time for SP resolution or SP treatment failure, which corroborates with the literature that suggests that resistant and susceptible infections may not have different treatment outcomes [7,31,32]. No bacterial isolate or resistance pattern at the enrollment were significant predictors for the time to SP resolution or recurrence. Although not statistically significant, the hazard ratio for SP recurrence was nearly two times higher for the *Staphylococcus* sp. + cocci + rod group at the enrollment compared to the *Staphylococcus*-only group. This finding suggests that mixed infections may contribute to a higher likelihood of SP recurrence. Interestingly, although not statistically significant, a multidrug-resistant status was associated with over three times higher hazard ratio for a faster SP resolution compared to XDR cases. We compared the susceptibility pattern of the dogs who experienced SP recurrence at enrollment and exit. Due to the small numbers in the resistance pattern groups at exit, further statistical analysis was not possible, and only the frequencies were tabulated.

Four dogs developed *Malassezia* dermatitis throughout the study while still presenting with non-resolved SP. These four dogs were considered treatment failures, and their cutaneous infections were subsequently addressed with topical therapy. The possible association of *Malassezia* dermatitis and previous antibiotic therapy has been reported in the literature [33]. This situation is also observed with bacterial otitis externa cases, in which *Malassezia* overgrowth is observed during treatment with topical antibiotics [34]. It is known that *Malassezia* infections are often secondary to atopic dermatitis [35]. Therefore, we speculate that the development of *Malassezia* dermatitis in the studied dogs may have been influenced not only by the systemic antibiotic treatment but also by the dogs’ underlying allergic disease or a combination of both.

The dogs’ pyoderma severity score did not correlate with the bacterial resistance pattern at the study enrollment. In other words, dogs with higher severity scores did not always present with resistant isolates, while some dogs with lower pyoderma scores had resistant isolates. These observations suggest that the severity of SP clinical presentation is not predictive of the resistance pattern of the bacterium strain associated with the infection. Numerous studies have identified an association between prior systemic antimicrobial treatment and the identification of bacterial resistant infections [6,9,36,37,38]. This association did not seem to occur in the studied population, as a large number of dogs that had previously been treated with oral antibiotics still presented with susceptible bacterial isolates. The analysis of risk factors that could have influenced bacteria susceptibility patterns was beyond the scope of this study.

The major role of primary underlying diseases in SP development and recurrence is supported by the identification of the same *Staphylococcus* strains and susceptibility phenotype from lesions and carriage sites of dogs with SP [39]. The main underlying diseases associated with SP in dogs are allergic dermatitis, endocrinopathies, and cornification disorders [40], with cAD being reportedly the most common. It is mandatory that the underlying disease is identified and addressed concurrently with the SP treatment. All studied dogs were being treated for cAD prior to enrollment. The allergic disease was controlled, and the same treatment protocol was maintained during the study duration. We did not observe any association between time to SP resolution and recurrence and type of cAD medication used, suggesting that the overall control of cAD has more influence in the SP outcome than the specific treatments used.

The study has a few limitations of consideration. The investigators were not blind to the SP treatment protocols, which could have introduced evaluation bias. The reliance on the dogs’ owners for medication administration could have resulted in potential treatment inconsistencies. A relatively small number of dogs in each group completed the study. Pruritus level and the extent and severity of cAD were not evaluated during the study. It is possible that variations in cAD severity during the study period could have influenced SP recurrence. However, the dogs had their cAD well controlled at enrollment and study end, making this reasoning less likely. Clinical resolution of SP was assessed only at scheduled study visits. Although resolution may have occurred between visits, clinician assessment was used to determine the date of resolution rather than owner-reported resolution. Additionally, because molecular typing of bacterial isolates was not performed, it was not possible to determine whether recurrent episodes of SP represented relapse of the original infection or reinfection with a different bacterial strain after successful treatment. Lastly, a longer follow-up period would have led to a more dependable evaluation of the probability for SP recurrence in each group.

Our study did not identify evidence that continuing antibiotics for seven days beyond clinical resolution reduced recurrence within the follow-up period. This can help support recommendations for antibiotics to be administered until SP lesions are no longer observed rather than seven days beyond clinical resolution, as the extended treatment does not seem to prevent recurrence of SP. The length of systemic antibiotic therapy should be tailored to the individual’s clinical response. Recheck examinations should be scheduled often to determine the shortest course required to achieve SP resolution.

## Figures and Tables

**Table 1:** The frequency of the prescribed systemic antibiotic based on aerobic culture results and the treatment of cAD by group and bacterial isolate at enrollment.

	Bacterial Isolate at Enrollment	Number of Dogs	Antibiotic Prescribed						
			Amox./ Clavulanic Acid	Cefalexin	Clindamycin	Marbofloxacin	Chloramphenicol	Trimethoprim Sulfonamide	Rifampicin
**Group A**	*Staphylococcus* sp.	23	3	9	6	1	2	0	2
	*Staphylococcus* sp. + cocci	3	1	0	0	0	0	1	1
	*Staphylococcus* sp. + rod	8	0	1	1	1	0	1	0
	*Staphylococcus* sp. + cocci + rod	3	0	0	1	0	1	1	0
		37	4	10	8	2	3	3	3
**Group B**	*Staphylococcus* sp.	19	3	6	8	0	2	0	0
	*Staphylococcus* sp. + cocci	1	0	0	0	0	0	1	0
	*Staphylococcus* sp. + rod	6	2	1	1	2	0	0	0
	*Staphylococcus* sp. + cocci + rod	4	1	0	1	0	1	1	0
		30	6	7	10	2	3	2	0
	**Bacterial isolate at enrollment**	**cAD treatment**							
		**oclacitinib**	**lokivetmab**	**ciclosporin**	**prednisone**	**none**	**ciclosporin + prednisone**		
**Group A**	*Staphylococcus* sp.	12	3	0	3	3	2		
	*Staphylococcus* sp. + cocci	2	0	1	0	0	0		
	*Staphylococcus* sp. + rod	4	1	0	3	0	0		
	*Staphylococcus* sp. + cocci + rod	1	1	0	0	1	0		
		19	5	1	6	4	2		
**Group B**	*Staphylococcus* sp.	10	2	0	4	2	1		
	*Staphylococcus* sp. + cocci	1	0	0	0	0	0		
	*Staphylococcus* sp. + rod	4	0	1	1	0	0		
	*Staphylococcus* sp. + cocci + rod	2	0	1	1	0	0		
		17	2	2	6	2	1		

amox. = amoxicillin; cAD = canine atopic dermatitis.

**Table 2:** The bacterial isolates and *Staphylococcus* sp. resistance patterns at recurrence by group, including group A (dogs treated for seven days beyond clinical superficial pyoderma resolution) and group B (dogs not treated beyond clinical superficial pyoderma resolution). MDR = multidrug resistant; XDR = extensively drug resistant.

**Group A**		
**Bacterial isolates at recurrence**	**Number of dogs** **(*n* = 13)**	**Percentage (%)**
*Staphylococcus* sp.	5	38.5%
*Staphylococcus* + cocci	2	15.4%
*Staphylococcus* + rod	4	30.8%
Rod	2	15.4%
***Staphylococcus* resistance patterns**	**Number of dogs** **(*n* = 11)**	**Percentage (%)**
MDR	5	45.5%
Susceptible	3	27.3%
XDR	3	27.3%
**Group B**		
**Bacterial isolates at recurrence**	**Number of dogs** **(*n* = 10)**	**Percentage (%)**
*Staphylococcus* sp.	9	90.0%
*Staphylococcus* + rod	1	10.0%
**Staphylococcus resistance patterns**	**Number of dogs** **(*n* = 10)**	**Percentage (%)**
MDR	2	20.0%
Susceptible	4	40.0%
XDR	4	40.0%

## Data Availability

The original contributions presented in this study are included in the article/Supplementary Materials. Further inquiries can be directed to the corresponding author.

## Supplementary Materials

The following supporting information can be downloaded at: https://www.mdpi.com/article/10.3390/vetsci13090880/s1, Table S1: The non-*Staphylococcus* bacteria isolated on aerobic culture of superficial pyoderma lesions, categorized according to their morphology. Table S2: The bacterial isolate and bacterial resistance pattern at the study enrollment and recurrence from dogs in group A (dogs treated one week beyond clinical superficial pyoderma resolution). MDR = multidrug resistant, MR = methicillin-resistant, S = susceptible, XDR = extensively drug-resistant, N/A = not applicable (no *Staphylococcus* isolated). Table S3: The bacterial isolate and bacterial resistance pattern at the study enrollment and recurrence from dogs in group B (treated until superficial pyoderma clinical resolution). MDR = multidrug resistant, MR = methicillin-resistant, S = susceptible, XDR = extensively drug-resistant, SP = superficial pyoderma. Table S4: The bacterial isolates and bacterial resistance patterns at enrollment by group for dogs that experienced treatment failure, treatment failure + *Malassezia* dermatitis, or withdrawal from the study. MDR = multidrug resistant, MR = methicillin-resistant, S = susceptible, XDR = extensively drug-resistant. Table S5: Group A (dogs treated for seven days beyond superficial pyoderma clinical resolution): days to clinical resolution, days free from recurrent superficial pyoderma until the end of the study, prescribed oral systemic antibiotic, and concurrent medications, including dosages and administration frequencies. SP—superficial pyoderma. Table S6: Dogs in group A that showed treatment failure (no clinical resolution of superficial pyoderma): days treated without clinical resolution, prescribed oral systemic antibiotic, and concurrent medications, including dosages and administration frequencies. SP—superficial pyoderma. Table S7: Group B (dogs treated until clinical resolution of superficial pyoderma): days to clinical resolution, days free from recurrent superficial pyoderma until the end of the study, prescribed oral systemic antibiotic, and concurrent medications, including dosages and administration frequencies. SP—superficial pyoderma. Table S8: Dogs in group B that showed treatment failure (no clinical resolution of superficial pyoderma): days treated without clinical resolution, prescribed oral systemic antibiotic, and concurrent medications, including dosages and administration frequencies. SP—superficial pyoderma.
