# Supplementary material for: Antibiotic Discontinuation at Clinical Resolution Versus Seven Additional Days for Canine Superficial Pyoderma: A Randomized Clinical Study
Source: Vet Sci. 2026 Aug 28;13(9):880. doi: 10.3390/vetsci13090880 (PMC13611872; doi:10.3390/vetsci13090880)
Supplement: Supplementary file 1 [file vetsci-13-00880-s001.zip › vetsci-4463047-supplementary.pdf]

Cocci-shaped bacteria
Streptococcus sp.
Enterococcus sp.
Moraxella sp.
**Rod-shaped bacteria**
Enterobacter sp.
Escherichia coli
Corynebacterium sp.
Pseudomonas sp.
Proteus sp.
Actinomyces sp.
Actinetobacter sp.
Pantoea sp.
Serratia sp.
Stenotrophomonas sp.

Supplemental Table S1: The non-*Staphylococcus* bacteria isolated on aerobic culture of superficial pyoderma lesions, categorized according to their morphology.

Group A	Bacteria Enrollment	Resistance Enrollment	Bacteria Recurrence	Resistance Recurrence
1	*Staphylococcus* sp.	MDR	*Staphylococcus* sp.	MDR
5	*Staphylococcus* sp. + cocci + rod	MDR	*Staphylococcus* sp. + rod	MDR
8	*Staphylococcus* sp.	S	*Staphylococcus* sp. + rod	MDR
16	*Staphylococcus* sp.	S	*Staphylococcus* sp.	S
22	*Staphylococcus* sp.	XDR	rod	N/A
25	*Staphylococcus* sp.	S	*Staphylococcus* sp. + cocci	XDR
27	*Staphylococcus* sp.	S	*Staphylococcus* sp. + cocci	XDR
28	*Staphylococcus* sp. + cocci	MDR	*Staphylococcus* sp. + rod	MDR
31	*Staphylococcus* sp.	S	*Staphylococcus* sp.	MDR
33	*Staphylococcus* sp. + rod	MDR	rod	N/A
38	*Staphylococcus* sp. + cocci	XDR	*Staphylococcus* sp.	XDR
40	*Staphylococcus* sp.	S	*Staphylococcus* sp. + rod	S

55	*Staphylococcus* sp.	MDR	*Staphylococcus* sp.	S

Supplemental Table S2: The bacterial isolate and bacterial resistance pattern at the study enrollment and recurrence from dogs in group A (dogs treated one week beyond clinical superficial pyoderma resolution). MDR = multidrug resistant, MR = methicillin-resistant, S = susceptible, XDR = extensively drug-resistant, N/A = not applicable (no *Staphylococcus* isolated).

Group B	Bacteria isolated at study enrollment	Resistance at enrollment	Bacteria isolated at SP recurrence	Resistance at recurrence
12	*Staphylococcus* sp.	XDR	*Staphylococcus* sp.	XDR
13	*Staphylococcus* sp.	S	*Staphylococcus* sp.	S
14	*Staphylococcus* sp.	S	*Staphylococcus* sp. + rod	MDR
19	*Staphylococcus* sp.	S	*Staphylococcus* sp.	S
26	*Staphylococcus* sp.	MDR	*Staphylococcus* sp.	MDR
35	*Staphylococcus* sp. + rod	S	*Staphylococcus* sp.	XDR
43	*Staphylococcus* sp. + rod	S	*Staphylococcus* sp.	XDR
46	*Staphylococcus* sp.	MDR	*Staphylococcus* sp.	S
57	*Staphylococcus* sp. + cocci + rod	S	*Staphylococcus* sp.	XDR
67	*Staphylococcus* sp.+ rod	S	*Staphylococcus* sp.	S

Supplemental Table S3: The bacterial isolate and bacterial resistance pattern at the study enrollment and recurrence from dogs in group B (treated until superficial pyoderma clinical resolution). MDR = multidrug resistant, MR = methicillin-resistant, S = susceptible, XDR = extensively drug-resistant, SP = superficial pyoderma.

Group A	Bacteria isolated at study enrollment	Resistance at enrollment	Treatment failure	Treatment failure + *Malassezia* dermatitis	Withdrawn
2	*Staphylococcus* sp. + rod	XDR			X
3	*Staphylococcus* sp.	XDR	X		
4	*Staphylococcus* sp.	XDR			X

6	*Staphylococcus* sp.	S			X
7	*Staphylococcus* sp.	S	X		
15	*Staphylococcus* sp.	S			X
17	*Staphylococcus* sp. + cocci + rod	S	X		
30	*Staphylococcus* sp.	S	X		
32	*Staphylococcus* sp.	S	X		
39	*Staphylococcus* sp.	XDR	X		
41	*Staphylococcus* sp.	S	X		
44	*Staphylococcus* sp. + rod	S	X		
45	*Staphylococcus* sp.	S	X		
51	*Staphylococcus* sp. + rod	S	X		
52	*Staphylococcus* sp. + rod	S	X		
62	*Staphylococcus* sp. + rod	S	X		
63	*Staphylococcus* sp.	S		X	
Group B	Bacteria isolated at study enrollment	Resistance at enrollment	Treatment failure	Treatment failure + *Malassezia* dermatitis	Withdrawn
10	*Staphylococcus* sp.	XDR			X
11	*Staphylococcus* sp.	S	X		
18	*Staphylococcus* sp.	S		X	
23	*Staphylococcus* sp.	S	X		
24	*Staphylococcus* sp.	S		X	
34	*Staphylococcus* sp.	S			X
36	*Staphylococcus* sp.	S	X		

42	*Staphylococcus* sp. + cocci + rod	XDR	X		
53	*Staphylococcus* sp. + cocci + rod	S	X		
54	*Staphylococcus* sp. + rod	S		X	
58	*Staphylococcus* sp.	MR	X		
64	*Staphylococcus* sp.	S			X

Supplemental Table S4: The bacterial isolates and bacterial resistance patterns at enrollment by group for dogs that experienced treatment failure, treatment failure + *Malassezia* dermatitis, or withdrawal from the study. MDR = multidrug resistant, MR = methicillin-resistant, S = susceptible, XDR = extensively drug-resistant.

Dogs	Days to SP resolution	Days to SP recurrence	Days without SP recurrence until study end (Day98)	Antibiotic prescribed	Dose administered	Concurrent medications	Dose concurrent medications
1	39	31		marbofloxacin	4.9 mg/kg q24h	none	N/A
5	14	14		clindamycin	11.2 mg/kg q12h	none	N/A
8	28	28		cefalexin	28.4 mg/kg q12h	oclacitinib	0.6 mg/kg q12h
16	28	71		amoxicillin/ clavulanic acid	19.3 mg/kg q12h	lokivetmab	2 mg/kg q 4weeks
20	26		70	cefalexin	28.8 mg/kg q12h	oclacitinib	0.61 mg/kg q24h
22	28	70		rifampicin	3.9 mg/kg q12h	none	N/A
25	28	35		cefalexin	30 mg/kg q12h	oclacitinib	0.64 mg/kg q24h
27	28	15		cefalexin	31 mg/kg q12h	oclacitinib	0.6 mg/kg q24h
28	29	35		trimethoprim sulfonamide	15.6 mg/kg q12h	oclacitinib	0.52 mg/kg q24h

29	14		84	clindamycin	10 mg/kg q12h	oclacitinib	0.52 mg/kg q24h
31	14	65		clindamycin	13 mg/kg q12h	oclacitinib	0.52 mg/kg q24h
33	10	45		trimethoprim sulfonamide	17.9 mg/kg q12h	lokivetmab	2 mg/kg q 4weeks
38	30	28		rifampicin	5 mg/kg q12h	oclacitinib	0.64 mg/kg q24h
40	28	64		cefalexin	36.5 mg/kg q12h	prednisone	1.2 mg/kg q24h
48	14		84	clindamycin		oclacitinib	0.67 mg/kg q24h
49	15		84	clindamycin	10 mg/kg q12h	oclacitinib	0.6 mg/kg q24h
55	14	88		amoxicillin/ clavulanic acid	16 mg/kg q12h	prednisone	0.75 mg/kg q24h
56	14		79	marbofloxaci n	5 mg/kg q24h	oclacitinib	0.6 mg/kg q24h
60	14		84	clindamycin	12.3 mg/kg q12h	oclacitinib	0.65 mg/kg q24h
65	14		84	clindamycin	9.7 mg/kg q12h	oclacitinib	0.6 mg/kg q24h

Supplemental Table S5: Group A (dogs treated for seven days beyond superficial pyoderma clinical resolution): days to clinical resolution, days free from recurrent superficial pyoderma until the end of the study, prescribed oral systemic antibiotic, and concurrent medications, including dosages and administration frequencies.
SP – superficial pyoderma.

Dogs	Treatment days with no SP resolution	Antibiotic prescribed	Dose administered	Concurrent medications	Dose concurrent medication
3	28	chloramphenicol	43.86 mg/kg q8h	ciclosporin + prednisone	5.3 mg/kg q24h (C) + 0.53 mg/kg q24h (P)
7	28	cefalexin	31.3 mg/kg q12h	none	N/A
17	29	chloramphenicol	54.3 mg/kg q8h	lokivetmab	2 mg/kg q 4week

30	28	amoxicillin/ clavulanic acid	16.3 mg/kg q12h	oclacitinib	0.52 mg/kg q24h
32	25	cefalexin	30 mg/kg q12h	lokivetmab	2 mg/kg q 4week
39	28	chloramphenicol	22 mg/kg q8h	oclacitinib	0.5 mg/kg q24h
41	31	clindamycin	12.5 mg/kg q12h	oclacitinib	0.65 mg/kg q24h
44	29	amoxicillin/ clavulanic acid	14.3 mg/kg q12h	ciclosporin + prednisone	2.55 mg/kg q24h (C) + 5.8 mg/kg q24h (K)
45	23	clindamycin	10 mg/kg q12h	lokivetmab	1.91 mg/kg q 4week
51	30	cefalexin	30.3 mg/kg q12h	prednisone	0.96 mg/kg q24h
52	30	clindamycin	10.1 mg/kg q12h	prednisone	0.9 mg/kg q24h
62	28	clindamycin	11 mg/kg q12h	prednisone	1 mg/kg q24h
63	28	cefalexin	30.5 mg/kg q12h	oclacitinib	0.65 mg/kg q24h

Supplemental Table S6: Dogs in group A that showed treatment failure (no clinical resolution of superficial pyoderma): days treated without clinical resolution, prescribed oral systemic antibiotic, and concurrent medications, including dosages and administration frequencies.
SP – superficial pyoderma.

Dog	Days to SP resolution	Days to SP recurrence	Days without SP recurrence until study end (Day98)	Antibiotic prescribed	Dose prescribed	Concurrent medications	Dose concurrent medications
9	28		70	trimethoprim sulfonamide	14.7 mg/kg q12h	oclacitinib	0.6 mg/kg q24h
12	14	14		chloramphenicol	46 mg/kg q8h	oclacitinib	0.55 mg/kg q24h
13	14	28		amoxicillin/ clavulanic acid	15 mg/kg q12h	oclacitinib	0.58 mg/kg q24h
14	14	14		cefalexin	30.8 mg/kg q12h	lokivetmab	2.5 mg/kg q 4weeks

19	14	70		cefalexin	30.1 mg/kg q12h	none	
21	14		83	cefalexin	31.1 mg/kg q12h	lokivetmab	2 mg/kg q 6weeks
26	29	69		clindamycin	8.8 mg/kg q12h	oclacitinib	0.63 mg/kg q24h
35	14	14		marbofloxacin	5 mg/kg q24h	oclacitinib	0.6 mg/kg q24h
37	40		63	cefalexin	30 mg/kg q12h	oclacitinib	0.65 mg/kg q24h
43	29	14		amoxicillin/ clavulanic acid	16.3 mg/kg q12h	ciclosporin+ ketoconazole	3.2 mg/kg q24h (C)+ 6.5 mg/kg q24h (K)
46	15	44		clindamycin	10.6 mg/kg q12h	oclacitinib	0.56 mg/kg q24h
47	15		80	clindamycin	12.4 mg/kg q12h	oclacitinib	0.55 mg/kg q24h
50	15		88	clindamycin	10.8 mg/kg q12h	oclacitinib	0.58 mg/kg q24h
57	28	14		trimethoprim sulfonamide	14.6 mg/kg q12h	oclacitinib	0.7 m/kg q24h
59	14		90	cefalexin	29.6 mg/kg q12h	oclacitinib	0.63 mg/kg q24
61	27		67	clindamycin	10 mg/kg q12h	oclacitinib	0.66 mg/kg q24h
66	28		72	amoxicillin/ clavulanic acid	16.1 mg/kg q12h	ciclosporin + ketoconazole	2.86 mg/kg q24h (each medication)
67	29	12		amoxicillin/ clavulanic acid	16.3 mg/kg q12h	oclacitinib	0.52 mg/kg q24h

Supplemental Table S7: Group B (dogs treated until clinical resolution of superficial pyoderma):days to clinical resolution, days free from recurrent superficial pyoderma until the end of the study, prescribed oral systemic antibiotic, and concurrent medications, including dosages and administration frequencies.
SP – superficial pyoderma.

Dogs	Treatment days with no SP resolution	Antibiotic prescribed	Dose administered	Concurrent medications	Dose concurrent medications
11	28	amoxicillin/ clavulanic acid	14.7 mg/kg q12h	oclacitinib	0.6 mg/kg q24h
18	28	cefalexin	32.8 mg/kg q12h	oclacitinib	0.65 mg/kg q24h
23	28	amoxicillin/ clavulanic acid	13.7 mg/kg q12h	prednisone	1.09 mg/kg q24
24	28	clindamycin	10.9 mg/kg q12h	prednisone	1.09 mg/kg q24
36	28	clindamycin	10 mg/kg q12h	ciclosporin + prednisone	5 mg/kg q24h (C)+0.48 mg/kg q48h (P)
42	35	chloramphenicol	46.1 mg/kg q8h	prednisone	0.96 mg/kg q24h
53	26	clindamycin	9.3 mg/kg q12h	oclacitinib	0.6 mg/kg q24h
54	29	marbofloxacin	3.8 mg/kg q24h	prednisone	0.75 mg/kg q24h
58	29	clindamycin	11 mg/kg q12h	oclacitinib	0.6 mg/kg q24h

Supplemental Table S8: Dogs in group B that showed treatment failure (no clinical resolution of superficial pyoderma): days treated without clinical resolution, prescribed oral systemic antibiotic, and concurrent medications, including dosages and administration frequencies.

SP – superficial pyoderma.
